# Lightning for Energy and Material Uses: A Structured Review

**DOI:** 10.1002/gch2.202000029

**Published:** 2020-08-05

**Authors:** Daniel S. Helman

**Affiliations:** ^1^ Education Division College of Micronesia‐FSM Yap Campus PO Box 286 Colonia Yap 96943 Federated States of Micronesia

**Keywords:** dusty plasma, high‐voltage phenomena, lightning energy, plasma arc processing, targeted lightning

## Abstract

The average atmospheric charge density of Earth is neutral. Charge built up from thunderstorms and lightning phenomena is offset by oceanic surface charging, and offers a source of energy that has not been harnessed broadly. Unfortunately, the total terrestrial energy of the Earth’s atmospheric electrical system is modest (250–500 MW) compared to industrial requirements: Innovations are likely to offer improvements to societal efficiency rather than broad transformations. Direct capture systems located in places with very high occurrence of lightning discharge can generate ≈1 kWh per year on average. Material processing via triggered lightning is limited to techniques that utilize rapid discharges, e.g., metal and glass preprocessing of materials, waste volume reduction, biomass energy conversion, where current prices make plasma‐arc processes prohibitive. Triggered lightning may be used to assist blasting of mountain rock; or as a high‐voltage input for processes such as nuclear fusion. Passive collection of atmospheric electricity is modest but may be used in urban agriculture to increase biomass production. Thunderstorm charge‐separation processes suggest a new class of electricity generators based on kinetic energy and material collision. Ball lightning suggests additional research in dusty plasmas. These methods are all at proof‐of‐concept or early translation stages.

## Graphical abstract

The article highlights several current techniques including passive energy harvesting systems and the use of supercapacitors, plus material processing, and applications for agriculture. The electrical potential from lightning phenomena does not offer sufficient energy for direct use even in locations with the highest lightning frequency, but passive capture may be of benefit, and lightning may be suitable for material processing.

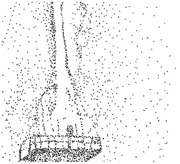

## 1 Background

**Figure 1:**
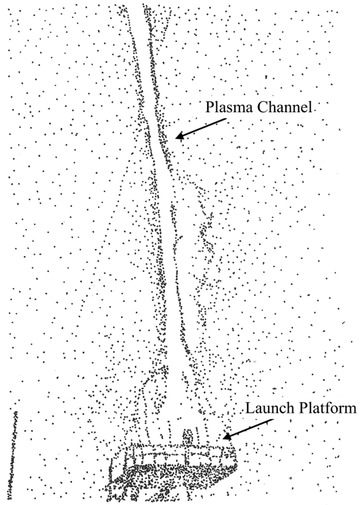
Rocket‐triggered lightning. Note that the plasma channel lacks the forked characteristic of natural lightning. Image redrawn from Dwyer et al.^[^ ^2b^ ^]^

This work is structured as a follow‐up to an earlier article related to catching lightning for energy,^[^ ^1^ ^]^ a review of what exists in the academic literature related to using a tower or rocket with a wire tether to guide a strike to earth, and then capture some part of its power with a buried inductor. Rocket triggering is a well‐established protocol for studying lightning parameters,^[^ ^2^ ^]^ but perhaps the power output of a single strike may not justify the expense for a rocket. In rocket‐triggered lightning, a dedicated platform is used to launch a rocket into a thundercloud. The rocket typically has a conducting wire attached that is vaporized on strike initiation, i.e., an exploding wire (see **Figure** **1**).

Lightning can be used for processing materials. Electric arcs have also been used to vitrify waste products, notably asbestos, in research at the US Army Corps of Engineers Construction Research Laboratories.^[^ ^3^ ^]^ The mineral fibers are turned to glass, and are no longer asbestiform, thus no longer asbestos nor a health risk. See **Table** **1** for a list of industrial and experimental uses of plasma arc technology identified in Zaghloul et al.^[^ ^3b^ ^]^ Notwithstanding, the power needed for the electric arc is high, e.g., 100 kW for several hours, depending on the waste stream.^[^ ^3b^ ^]^ The idea that lightning could be a source for the electric arc for this process seems a useful hypothesis, and the topic of this structured review includes lightning for both energy and material uses.

**Table 1:** Industrial and experimental uses of plasma arc technology (after Zaghloul et al.)^[^ ^3b^ ^]^

Can use plasma arc technology
Titanium scrap melting
Coal gasification
Ferroalloy production
Molten steel ladle heater
Aluminum recovery from dross
Volume reduction of equipment
Tundish heating for steel casting
Iron ore reduction
Biomass energy conversion
Shale oil recovery
Zinc recovery
Chemical synthesis
MgO refractory production
Powder metal production
Silicon metal production
Incinerator ash vitrification
Electric arc furnace dust vitrification
Glass melting
Waste pyrolysis
• municipal
• medical
• asbestos
• tires
• hazardous/toxic
• low‐level radioactive

## 2 Results and Analysis

The most common results were for projects where lightning phenomena and frequency are used as predictors for other events, e.g., to predict rainfall, flash floods, fires, volcanic processes, climate change occurrence, or variations in the El Niño Southern Oscillation (ENSO). These analyses are well‐developed areas of research. It was felt that these were fundamentally different from what the project was aiming for, and thus the use of lighting in prediction studies is not included. Rather, the focus remains on energy or material (physical) uses of lightning phenomena.

More than 100 relevant authors were found. Their work was organized into the following topics relevant to lightning for energy or material uses: cloud physics; lightning physics; atmospheric electricity; lightning protection; lightning direct and inductive capture; ball lightning; electric discharge into water; artificial lightning; conductors; supercapacitors; energy harvesting; plasma physics; nuclear fusion (general); nuclear fusion from lightning; lightning and dark matter; material effects of lightning strikes; lightning to process waste; lightning and biology; geomorphology; archaeology and lightning; and lightning and art. See **Table** **2** for the tally of authors writing in each category as well as tallies of interest in submission to a journal special issue on the topic. Twenty‐two (22) out of 116 authors responded to express their interest, i.e., a rate of approximately 20%. Three out of the twenty‐two authors submitted manuscripts, for a submission rate of about 15% for those who had expressed an interest; with an overall submission rate of about 2.5% of those initially contacted. See the Supplemental File for the search notes, including the names and affiliations of researchers, plus a description of their work and a sample reference.

**Table 2:** Results of searches for submissions on lightning for energy and material uses

Topic	Authors	Interested	Submissions
Cloud physics	1		
Lightning physics	2		
Atmospheric electricity	5	1	
Lightning protection	1		
Lightning direct and inductive capture	22	6	
Ball lightning	5	1	
Electric discharge into water	6	1	
Artificial lightning	1		
Conductors	2		
Supercapacitors	8	1	
Energy harvesting	5	1	
Plasma physics	5	3	1
Nuclear fusion (general)	2		
Nuclear fusion from lightning	7	2	
Lightning and dark matter	1		
Material effects of lightning strikes	15	1	1
Lightning to process waste	6		
Lightning and biology	14	2	
Geomorphology	2		
Archaeology and lightning	4	2	1
Lightning and art	2	1	
Total	116	22	3

Launching a special issue was not successful. Only three manuscripts were received after nearly a year of contacting potential authors, and only two of these were deemed suitable for peer‐review. The author corresponded with 17 other researchers who expressed an initial interest but did not ultimately submit papers. In total, 22 authors had expressed some interest; three of these were part of the same lab. The results of what was found in surveying the research to gather ideas for a special issue may be of interest to others working in this field or hoping to; thus this review article might be of some use. This Results and Analysis section describes the areas of research that had been uncovered in the author search. These are far‐reaching and their description comprises the second period of this project, i.e., synthesizing and communicating the results. Each topic in Table 2 is described in a separate subsection below, each providing a clear description of how the area is relevant to the use of lightning for energy or material uses. Expanded descriptions of some topics are given later in the Discussion section.

### 2.1 Cloud Physics

The Earth’s surface (including oceans (3.2 S m^−1^) and land (10^−7^–10^−2^ S m^−1^) and the ionosphere (10^−7^ S m^−1^) are electrically conductive, and are separated by atmosphere which acts as an electrical insulator (2 × 10^−15^–2 × 10^−14^ S m^−1^).^[^ ^4^ ^]^ Atmospheric cloud processes include charge separation within clouds, and are responsible for the electronic structure of thunderstorm clouds which allow for lightning discharge. See **Table** **3** for a summary from Saunders detailing observations and conclusions about electric charging of thunderstorm clouds.^[^ ^5^ ^]^ Mason is preeminent in his work on cloud physics and meteorology.^[^ ^6^ ^]^ Correspondence was not answered.

**Table 3:** Electric charge development in thunderstorms

Framework	Information
Observations	Negative charge region at 7 km corresponding to −15 °C Positive charge centers rise at updraught speed Increases in electric field strength in regions containing liquid water and ice particles Regions with strong electric field associated with ice crystals and graupel pellets Electrification occurs at updraught/downdraught interface
Conclusions	Charging process related to precipitation Charging mechanism: Ice crystals rebound from riming graupel in the presence of supercooled liquid water Additional charging via induction

### 2.2 Lightning Physics

**Figure 2:**
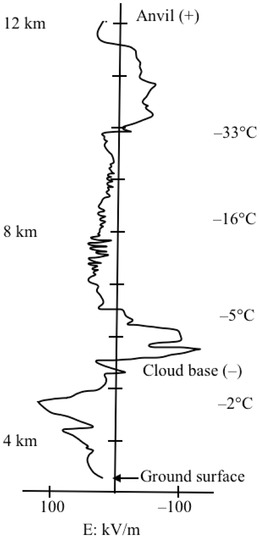
Observed electric field via balloon assay in a thundercloud by height. Note the cloud’s negative base and the positive anvil, as well as the temperatures. Image data from Winn et al.^[^ ^8b^ ^]^

Understanding the processes whereby lightning phenomena are triggered, leading to the electronic breakdown of the plasma channel, as well as its intensity and duration, have benefited from study of the phenomena themselves, as well as with rocket‐triggered launches since the 1960s. While there are many researchers working on lightning physics,^[^ ^7^ ^]^ the two identified in this search either declined to participate or did not respond.^[^ ^8^ ^]^ See **Figure** **2** for observed electric field variation by height in a thundercloud.^[^ ^8b^ ^]^

### 2.3 Atmospheric Electricity

The average atmospheric charge density of the Earth is neutral. Nevertheless, the charge built up from thunderstorms and lightning phenomena is offset by oceanic surface charging,^[^ ^9^ ^]^ and offers an attractive source of energy that has not been harnessed broadly. The global atmospheric electric circuit at any given time has a total current of ≈1–2 kA and a potential of ≈250 kV for a total power of 250–500 MW.^[^ ^10^ ^]^ The total current is based on estimates of thunderstorm activity and precipitation driving negative charge downward, plus the geomagnetic field and solar wind interactions. Thunderstorm atmospheric charging is offset by fair weather electrical currents (≈2 pA m^−2^). The potential of ≈+250 kV of the ionosphere compared to the ground is the result of the action of thunderstorms driving positive charges upward.^[^ ^10^ ^]^

The total power of the atmospheric electric circuit (250–500 MW) can be thought of as based on natural electricity consumption in the global atmospheric circuit rather than on natural global capacity. The author is not aware of any studies nor experiments clarifying the relationship between natural consumption and natural capacity. For comparison, total global electricity generation capacity of human civilization in 2014 was 6.142 TW.^[^ ^11^ ^]^

Two of the five researchers found during this project search and who study usage of atmospheric electricity write about energy capture of atmospheric electricity during storm activity.^[^ ^12^ ^]^ The experiments of one, Ariza González, relate to corona‐current capture. Corona current arises from the combination of wind and an electric field in the air. Capture is achieved with a bank of capacitors connected to a 40 m tall tower fitted with a needle electrode, and with an intervening surge protector and grounding so that strikes and overvoltages are protected against. See **Table** **4** for specifications. His proof‐of‐concept experiments produced positive results, i.e., ≈30 J per storm for five co‐located electrodes at 21 m above ground. Experiments ended with equipment failure from overvoltage. Plans for an array of corona towers for atmospheric electricity use are being investigated further by the Administrative Department of Science in Colombia (COLCIENCIAS), yet the yield is predicted to be small, about 6 kJ (1.67 Wh) per thunderstorm for an array of electrodes on 100 corona towers.^[^ ^12b^ ^]^

**Table 4:** Specifications of the Ariza González Corona‐Capture Tower

Component	Specification
Electrode	Needle tip: 0.1 m length, 0.4 mm diameter Rod: 0.7 m length, 14.3 mm diameter Material: copper
Tower	Steel tension Experimental: 21 m from ground Planned: 40 m from ground
Conductors	Coaxial Inner: copper, 2.5 mm radius, 1.7 × 10^−8^ Ω m resistivity Outer: steel, 25.4 mm external radius, 1.5 mm thick, 30 × 10^−8^ Ω m resistivity, 2000 relative permeability Outer conductor connected to tower and ground for lightning protection
Capacitor bank	Capacitor types: 1 nF, 0.47 μF, 47 μF, and 1000 μF Experimental working voltage: 400 V Planned working voltage: 10 kV
Surge protection	Air gap to ground–inductor–air gap to ground Inductor (1.5 μH) prevents surge Dischargers: 8 mm diameter, 4 mm length point, 1 mm air gap Coaxial cable: electrode–surge protection–capacitor bank Metal shielding for skin effect Tower cable: covered by 50.8 mm (2 in) wide tube Discharger 1: covered by 0.5 m wide box Inductor: covered by 0.5 m wide box Discharger 2 + capacitors: covered by 0.5 m wide box Offset between boxes: 0.5 m

Breuer looks at the atmospheric system as a whole and suggests that the average charge density is very low, and that capture would be limited by the need to move devices to follow charge densities.^[^ ^13^ ^]^ Yet Ogram describes a conductive tether system that can charge an aircraft or weather balloon, such that devices could indeed follow charge densities.^[^ ^14^ ^]^ Notwithstanding, his analysis does not include the energy consumption of the vehicle. One may consider that this type of device might be a passive energy harvesting system similar to the braking systems in certain cars that helps them to increase fuel efficiency.

Historically, smoke is correlated with increased atmospheric potential gradient in urban settings. Smoke and other aerosol particles can hold electric charge. Harrison writes about fair weather atmospheric electricity and air pollution.^[^ ^15^ ^]^ The change in potential gradient locally is correlated with air pollution. He does not suggest harvesting of this potential, but rather using it for reconstructing past conditions or present monitoring. He includes data related to the potential gradient (PG) under different weather conditions, and these are listed in **Table** **5**.

**Table 5:** Potential gradient (PG) under different weather conditions at one location (Reading, UK, 2010)

Weather conditions^a^ ^)^	Median PG [V m^−1^]	PG IQR^b^ ^)^ [V m^−1^]
Snow	191.6	48.7
Fog	170.6	142.05
Clear sky	93.5	43.44
Broken clouds, dry	91.1	41.68
Overcast, dry	81.4	44.65
Heavy rain	−4.9	179.96
All conditions^c^ ^)^	80.6	42.9

a ^)^Annual data (8447 h)

b ^)^IQR: inter‐quartile range (75%ile–25%ile)

c ^)^“All conditions” includes data from other weather categories not presented.^[^ ^15^ ^]^

### 2.4 Lightning Protection

**Figure 3:**
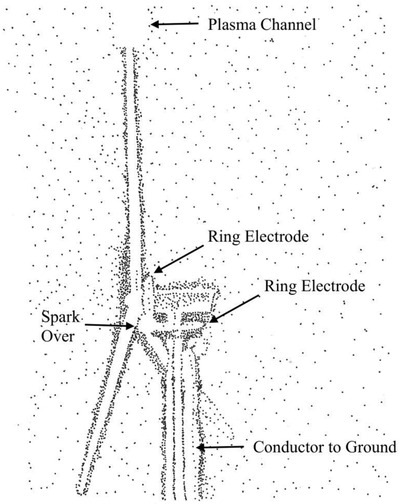
Operation of ring electrodes on a wind turbine (1:100 scale model). Image redrawn based on the work of Yoh.^[^ ^16^ ^]^

Infrastructure protection from lightning includes devices such as horns that help to prevent strikes on structures, and arresters for transmission lines that help to open and close circuits in the case of overvoltages. More recently, technology to use wind energy has necessitated the invention of ring conductors to protect wind power generators. Ring conductors placed near the base of the blades and on the structure’s shaft allow for a conduction of charge to the ground while the blades are spinning. See **Figure** **3** for an example of spark‐over between ring electrodes.

For any structure, integrating ambient energy capture with a lightning protection system is conceptually possible, but presents a design conflict between two goals: protection from lightning and energy production from it. Lightning protection takes precedence. Cetin is the lead author on a study of lightning protection for buildings optimized for renewable energy.^[^ ^17^ ^]^ Lightning protection is a very well‐developed field of study, but is not integrated with capture.

### 2.5 Lightning Direct and Inductive Capture

Lightning flashes consist of a series of strokes with microsecond to 0.1 µs durations.^[^ ^7a^ ^]^ The strokes alternate electric polarity as they bring charge to and from cloud to ground. The series of strokes has an approximate total of 0.5 s duration. Thus, direct capture of lightning electricity ought to be robust enough to transmit very large power over a very short time, and allow for very rapid switching of polarity.

Experimental results of a small scale model lightning capture system making use of a capacitor and high speed switching is found in Basar et al.^[^ ^18^ ^]^ In 2010, they describe a model system using capacitors to harness lightning.^[^ ^19^ ^]^ A patent also makes use of direct capture using capacitors,^[^ ^20^ ^]^ but it is not clear whether any data have been generated or the device used.

Another several papers summarize the possibilities for lightning power capture or propose techniques but these are conceptual rather than experimental.^[^ ^1^, ^21^ ^]^ Another paper found reports on an AC energy scavenging technique using magnets adjacent to a conductor.^[^ ^22^ ^]^ The data are not related to lightning per se.

Palmer used simulated lightning to charge a cell‐phone in 2013, but a large‐scale system has not been built.^[^ ^23^ ^]^ Likewise, Strahm runs a company (Alternative Energy Holdings Inc.) that aimed to harness lightning power, but the work does not seem to have been successful.^[^ ^24^ ^]^ Vlastic ran a team of architects and designers who entered a lightning‐harvesting skyscraper in an architectural competition, but the details of how it would work were not included.^[^ ^25^ ^]^ It was designed without a working concept. **Table** **6** summarizes the results of this section and includes information on type of method for lightning power use and whether data are associated with it.

**Table 6:** Some methods for lightning power use

Method	Author(s)	Content
Capacitors	Lai 2014^[^ ^20^ ^]^ Toohie et al., 2013^[^ ^21f^ ^]^ Basar et al. 2011^[^ ^18^ ^]^ Helman, 2011^[^ ^1^ ^]^ Basar et al. 2010^[^ ^19^ ^]^ Bhattacharjee, 2010^[^ ^21c^ ^]^	Patent Concept Laboratory data Concept Laboratory data Concept
Storage battery	Bhattacharjee, 2010^[^ ^21c^ ^]^	Concept
Transformer stepdown	Palmer (University of Southampton, 2013)^[^ ^23^ ^]^	Laboratory data
Laser trigger	Kalair et al., 2013^[^ ^21d^ ^]^	Concept
Inductor	Paprotny et al., 2013^[^ ^22^ ^]^ Helman, 2011^[^ ^1^ ^]^	Laboratory data Concept
Heat transducer	Toohie et al., 2013^[^ ^21f^ ^]^ Bhattacharjee, 2010^[^ ^21c^ ^]^	Concept Concept
Mechanical transducer	Malavika and Vishal, 2013^[^ ^21e^ ^]^ Bhattacharjee, 2010^[^ ^21c^ ^]^	Concept Concept
Piezoelectric: thunder	Muller, 2013^[^ ^21a^ ^]^	Concept
No method	Szczykulska et al., 2013^[^ ^21b^ ^]^ Vlastic (Furuto, 2011)^[^ ^25^ ^]^	Concept Concept
Unknown	Strahm (Bloomberg, 2018)^[^ ^24^ ^]^	Unknown

### 2.6 Ball Lightning

This form of lightning discharge is rare in nature, and has been recorded with modern instrumentation only recently.^[^ ^26^ ^]^ Reports show variations in size, color and duration (1–10 s), with presence inside structures suggesting a relation to radio waves for its stability based on resonance, and presence close to the ground suggesting a soil‐lightning mechanism. The spectra reported are consistent with a mechanism whereby the phenomenon is caused by filamentary networks ejected from the soil after a lightning strike. See **Table** **7** for some features of ball lightning described in Bychkov et al.^[^ ^27^ ^]^

**Table 7:** Some features of ball lightning from Bychkov et al^[^ ^27^ ^]^

Some ball lightning features
Energy density: 10^10^–10^12^ J m^−3^ (estimated from water interactions)
Some reported symptoms consistent with radiation sickness
Can appear after linear lightning discharge in the channel, cloud, earth, or on metallic conductors
Incorporate inorganic or organic particles, dust, soil, water, or other materials
Structure: internal features (luminescent grains) and a vitrified cover are sometimes observed
Laboratory experiments and modeling consistent with initiation by linear lightning via:
Cavitation
Plasma circulation
Free charge in central region
Steady state from fusion reactions between light nuclei in
a core region

The stability and dynamics of ball lightning are of interest for plasma studies. Four of the five authors found on this theme describe the mechanics of ball lightning.^[^ ^27^, ^28^ ^]^ Abrahamson and Dinniss also write about the changes to soil in addition during a ball lightning strike.^[^ ^29^ ^]^ Their work formed the basis for the physical mechanism described by Cen et al.^[^ ^26^ ^]^

### 2.7 Electric Discharge into Water

**Figure 4:**
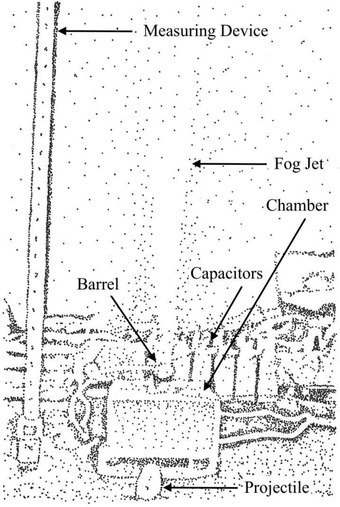
Measurement of kinetic energy from electric arc explosions in water. Projectile at rest after explosion. Image redrawn based on the work of Leavitt.^[^ ^30^ ^]^

Lightning discharge into water is explosive. Harnessing the resulting kinetic energy is possible. Leavitt is a student hobbyist, experimenting with the kinetic energy available from explosions in water caused by electric arcs.^[^ ^30^ ^]^ His research work includes experimental data gathered by measuring the propulsion of a projectile. See **Figure** **4**. This work suggests that lightning may be directed to a water‐filled chamber, with a resulting steam explosion turning a turbine within an escape channel may be possible. Leavitt’s data show kinetic energy in excess of input energy, arising from an error or some internal process of indeterminate origin. Although unrelated to energy capture, Anpilov et al. give a detailed description of how conductivity changes during discharge into water.^[^ ^31^ ^]^

**Figure 5:**
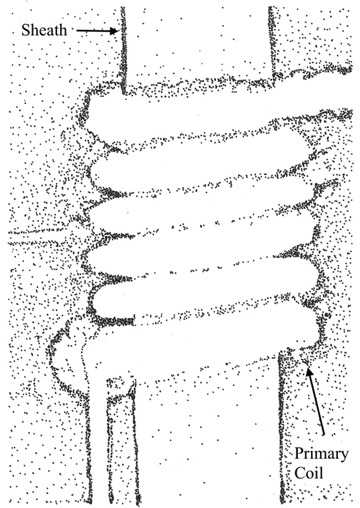
Transducer with plasma primary coil. Secondary coil and air core are inside a heat resistant sheath. Image redrawn based on the work of Sinton et al.^[^ ^39a^ ^]^

### 2.8 Artificial Lightning

Research is commonly carried out to create electric discharge that approximates lightning phenomena so that devices or other technology may be tested for lightning protection efficacy. Walko was a principal investigator in studies related to artificial lightning generation for testing purposes.^[^ ^32^ ^]^ Correspondence was not answered.

### 2.9 Conductors

Conductance is the inverse of resistance. Increased conductance can change the feasibility of power production. For example, the recent development of high temperature superconductors may make fusion energy feasible in the nearterm, as losses prevent parity. Alternately, if lightning energy is harvested by buried inductors, as has been suggested by the author,^[^ ^1^ ^]^ or if the lightning protection of buildings is to include capacitors to capture some of the charge transmitted to the ground, improved conductors will be important. Patz and Davenport have written patents on highly conductive fibers and materials that may be useful for harnessing lightning.^[^ ^33^ ^]^ Their company policy (Hexcel Corporation) prevented them from sharing any more information about their work or participating in the project.

### 2.10 Supercapacitors

Eight authors who work with supercapacitors and ultracapacitors were contacted.^[^ ^34^ ^]^ Kularatna responded positively, with a plan to implement some experiments connecting ultracapacitors to part of a structure’s lightning protection system. It is not known whether these were undertaken, but he is active in supercapacitor design for sustainable energy applications.^[^ ^35^ ^]^ See **Table** **8** for a comparison of supercapacitor and typical electrolytic capacitor properties.

**Table 8:** Comparison of properties of typical electrolytic capacitors and supercapacitors^[^ ^35^ ^]^

Property	Capacitor	Supercapacitor
Capacitance [F]	10^−12^–10^−3^	0.1–5000
Power density [W kg^−1^]	10^7^	3000
Energy density [Wh kg^−1^]	0.1	3
Time of charge/discharge [s]	10^−6^–10^−3^	0.3–30
Cyclability	10^10^	10^6^
Efficiency [%]	>95	85–98
Typical lifetime [yrs]	30	30

### 2.11 Energy Harvesting

Energy harvesting as a field looks at modest amounts of energy stored for low energy applications, for example, in wearable electronics. Three of the five authors found in this category are coauthors on a single paper related to alternating current (AC) energy capture in systems with mechanical or radiofrequency (RF) transducers.^[^ ^36^ ^]^ Another also works with RF capture.^[^ ^37^ ^]^ And another describes energy harvesting as it relates to smart systems but is not working on lightning per se except on a sensor array for detection.^[^ ^38^ ^]^ The author found no work being carried out matching lightning energy with energy harvesting.

### 2.12 Plasma Physics

Lightning strikes are plasma phenomena, i.e., the dielectric breakdown of air forms a plasma channel. Capturing energy from lightning may require new techniques for working with plasmas. One group has been working on plasma phenomena and exploding wires—and formed a private company to further their research.^[^ ^39^ ^]^ One of their research ideas is to adapt the geometry of the exploding wires for various applications including very high voltage transformers. If they are successful, their work would lend itself to providing a plasma source or magnetic field technology for commercial fusion. Lightning harvesting for these functions could be an extension of their work. Likewise, other researchers are doing relevant work: Zhukov works in plasmas and solid state materials.^[^ ^40^ ^]^ Schwabe works in dusty plasmas.^[^ ^41^ ^]^

### 2.13 Nuclear Fusion (General)

Using nuclear fusion of heavier elements from lighter elements requires a large amount of initial energy. Sourcing that energy from lightning phenomena may make the process feasible. Winterberg has written about fusion reactions of deuterium with gigavolt generators, whose characteristics he compares explicitly to lightning (though he does not suggest using lightning).^[^ ^42^ ^]^ Nakai works in laser triggering of fusion reactions.^[^ ^43^ ^]^ Neither author responded to queries.

### 2.14 Nuclear Fusion from Lightning

As described above, Winterberg argues for a method of generating high voltages to fuse deuterium that will mimic natural fusion reactions in lightning.^[^ ^42^ ^]^ In fact, lightning phenomena are associated with neutron flux that may be consistent with nuclear fusion of light elements. Both Gurevich et al. and Chilingarian et al. write about neutron generation, i.e., a strong flux of low‐energy neutrons during thunderstorm activity.^[^ ^44^ ^]^ Shah et al. measure 2.45 MeV (i.e., consistent with deuteron–deuteron fusion) neutron flux within 320 µs of lightning strokes.^[^ ^45^ ^]^ Comparing these to manually triggered neutron counts in **Table** **9** shows some evidence of fusion events.

**Table 9:** Experimental results showing neutron‐generation events (bold) consistent with deuteron–deuteron fusion in lightning

No. of neutrons in event	Control	Lightning
1	8299 (98.84%)	10 818 (96.65%)
2	97 (1.11%)	**250 (2.23%)**
3	4 (0.05%)	**40 (0.35%)**
>3	0 (0.00%)	**84 (0.77%)**

Separately, four other authors write about the possibility of using the mechanics of ball lightning for sustained fusion reactions without magnetic containment.^[^ ^46^ ^]^ All write concept papers that affirm the possibility. Experimental details are not yet present.

### 2.15 Lightning and Dark Matter

Drobyshevski has written extensively on cosmological subjects and dark matter, including work which examines the possible role of electrically‐charged dark matter in mediating fusion reactions in ball lightning.^[^ ^47^ ^]^ The work is uncited and unconfirmed. Correspondence was not answered.

### 2.16 Material Effects of Lightning Strikes

If lightning can be used in the place of plasma arcs for some industrial processes, such as vitrification of materials for safe storage, or for creating highly reduced compounds, energy savings may be realized. For a summary of these processes, see Table 1. Fiske at al. look at natural shocks to create engineering materials.^[^ ^48^ ^]^ Their work is in relation to an engineering materials company for profit. Yang et al. write about the characteristics of lightning and also how it affects metal it strikes.^[^ ^49^ ^]^ Jones et al. look at the reducing effects of lightning striking oxides;^[^ ^50^ ^]^ Glindemann et al. have written about the effect of lightning strikes on the formation of reduced‐phosphorus chemical compounds.^[^ ^51^ ^]^

Several authors look at the effect of carbon being struck. One research group found examines the effects of using artificial lightning in coal beds and other high‐carbon rocks for the creation of fullerenes.^[^ ^52^ ^]^ No significant amount of fullerenes are found; nor are they common in nature, despite some positive reports;^[^ ^53^ ^]^ Daly et al. report finding fullerenes in about 1 in 100 fulgurites.^[^ ^54^ ^]^ Nix suggests using lightning to electrify coal beds and provide the energy for hydrocarbon processing.^[^ ^55^ ^]^ Trout et al. look at a graphite application and examine how it behaves during lightning phenomena.^[^ ^56^ ^]^

Other materials explored include polymers and special coatings;^[^ ^57^ ^]^ plus the deleterious effects of lightning strikes on concrete in buildings.^[^ ^58^ ^]^

### 2.17 Lightning to Process Waste

Zaghloul et al. were involved in a project that melted asbestos (and other hazardous materials) with plasma‐arc discharge.^[^ ^3b^ ^]^ After the application, this material is no longer asbestiform—and thus is no longer poses a health hazard. The danger in asbestos arises from the form. Asbestos is a class of minerals, and they are inert when not filamentous. See **Table** **10** for the operational parameters and results of the asbestos remediation project described by Zaghloul et al.^[^ ^3b^ ^]^ Circeo set up a private firm (Applied Plasma Arc Technologies) that specializes in this process.^[^ ^59^ ^]^ Pavlus writes about using plasma to process waste and generate power quoting Circeo.^[^ ^60^ ^]^ Likewise, López‐Callejas et al. write about treatment of materials and organisms in the medical sector using plasma‐arc application.^[^ ^61^ ^]^ None of these researchers suggest using natural lightning to provide a plasma arc.

**Table 10:** Abatement of asbestos‐containing material (ACM) with plasma arc^[^ ^3b^ ^]^

Trial	1	2	3	4
Sample parameters				
No. of canisters	3	5	6	6
Sample type	Floor tile	Transit panel	Roofing tile	Floor tile
ACM weight [g]	2141	2240	3120	2716
Total weight [g]	2600	3744	3949	3562
Operational data				
Preheat time [min]	60	45	42	42
Processing time [min]	15	24	30	30
Post‐processing time [min]	28	0	13	18
Total heating time [min]	103	69	85	90
Max. process temp. [°C]	1804	1843	2260	1985
Min. process temp. [°C]	1539	1615	1510	1425
Final melt temp. [°C]	942	942	1326	948
Torch/system conditions				
Average power [kW]	76.3	87.5	86.3	86.5
Average torch pressure [psi]	53.9	50.5	53.1	59.7
Torch height above crucible Preheat Processing	15ʺ 12ʺ–9ʺ–12ʺ	9ʺ 12ʺ	15ʺ 12ʺ–9ʺ	9ʺ 12ʺ–9ʺ
Vacuum (in. of water)	3.5	3.0	4.0	3.5
Steel melt [g]	2220	3330	None	None
Exhaust analysis				
NO_x_ [ppm]	>5000	>5000	>5000	>5000
CO [ppm]	1000	300‐500	100	>3000
HF [ppm]	<1.5	n/a	n/a	>7.5
H_2_S [ppm]	<1.0	n/a	<2.0	<1.0
Product analysis				
Recovery fraction	0.947	1.011	0.807	0.690

### 2.18 Lightning and Biology

Storm activity changes the ambient electrical energy at a site, and this energy is sometimes useful for ecosystem functions. One research group has written about electricity applied to a submerged medium promoting biomass production of a specific mushroom;^[^ ^62^ ^]^ similarly, Takaki et al. found that pulsed electricity applied to the growing area increases mushroom yield.^[^ ^63^ ^]^ The electricity may work by increasing the bioavailability of nutrients. In fact, another research group explored the effect of lightning on metal mobilization and its increased bioavailability after a strike;^[^ ^64^ ^]^ and another describes the mobilization of phosphorus for ecosystem use after a strike.^[^ ^65^ ^]^ Two other research groups have written about lightning and evolution, following on the Urey‐Miller experiments.^[^ ^66^ ^]^ Application of atmospheric electricity either in small amounts for increasing agricultural yields or as high voltage events for reduction reactions for soil amendment are implied by the above. See **Table** **11** for a detailed summary of this work.

**Table 11:** Recent research on biophysical effects of lightning

Topic	Mode of study	Effect	Author(s)
Metal mobilization and biomass availability	Soil pore water measurement after artificial lightning vs control	Fe: no effect Mn: Increased mobility: mean 0.025 mmol (negative strike) and 0.08 mmol (positive strike)	Schaller et al., 2013^[^ ^64^ ^]^
Phosphate reduction	Analyzed 10 fulgurites	Carbon‐rich: 22% phosphate reduced as iron phosphide Others: 37–68% reduced as phosphite	Pasek and Block, 2009^[^ ^65^ ^]^
Effects on fungi	Electricity applied to submerged medium	*Ganoderma lucidum*: Promotes biomass production Highest increase: AC electric field of 1.5 kV cm^−1^ applied at day 3: 35.6450 ± 0.6684 g l^−1^ Lowest increase: DC electric field of 2.5 kV cm^−1^ applied at day 6: 26.2950 ± 3.9926 g l^−1^ Intermediate: other arrangements of AC, DC, or pulsed electricity up to 3.0 kV cm^−1^; days 0, 3, or 6.	Ramírez‐Cadavid et al., 2010^[^ ^62^ ^]^
	Pulsed electricity: 50 kV 50 ns pulsed electricity (50 pulses) or 50–125 kV (single pulse) applied to growing medium natural/artificial log	*Lyophyllum decastes*, *Lentinula edodes*, *Pholiota nameko*, and *Naematoloma sublateritium*: increased mushroom yield by 1.5–2.1	Takaki et al., 2009^[^ ^63^ ^]^
Role in evolution	Hypothesis and review of literature	Horizontal gene transfer in prokaryotes via electroporation and electrofusion	Kotnik, 2013^[^ ^66a^ ^]^
	Hypothesis	Lightning for nitrogen oxides and ozone in the prebiotic Earth	Russell, 2007^[^ ^66b^ ^]^

### 2.19 Geomorphology

Strong magnetic fields can weaken materials, including earth materials. Lightning strikes as well can reduce materials strength. Knight and Wakasa have each done work related to lightning‐strike effects on landforms trying to quantify the effects, i.e., to show a quantifiable relation between lightning and concomitant rock weathering.^[^ ^67^ ^]^

### 2.20 Archaeology and Lightning

Geomagnetic surveys can be used to locate new archaeological sites. The remanent magnetism present is a category of thermoremanent magnetization of fire pits, ovens, hearths and similar functional areas, or sometimes the results of lightning strikes on buildings and other tall structures that are no longer extant.

The four authors found in this area do research related to the archaeological provenance of remanent magnetization, generally used to locate new sites to study.^[^ ^68^ ^]^ Maki et al. submitted a manuscript on burial mounds that may have been designed in such a way as to be struck. The presumption is that there is a cultural or religious significance and an aesthetic import to the effect of seeing these strikes repeatedly for the people who made the mounds. Their work was subsequently published in *The Minnesota Archaeologist*.^[^ ^69^ ^]^

### 2.21 Lightning and Art

Lightning has an aesthetic appeal. Likewise, because electricity can alter materials, lightning can be used as a medium for creating works of art. Johnson, who has been a research fellow at Fermilab, has used the accelerator to make art;^[^ ^70^ ^]^ he declined to be involved in this project. Ramesh, who has written about accidental images created during lightning discharge, showed an initial interest.^[^ ^71^ ^]^

## 3 Discussion

This section highlights some of the work that was found related to lightning for energy and material uses. It is a synthesis and extension of the previous section, and provides practical findings for whether lightning phenomena feasibly can be applied to energy or material uses.

### 3.1 Capture of Atmospheric Electricity

The total power of the Earth’s atmospheric electric circuit is about 250–500 MW. This is not enough power to justify large‐scale use of atmospheric electricity. A global power capture system with 100% efficiency running constantly might at its maximum capture 1 TWh each year. Actual global yields of 1 GWh from a robust global system are more in line with currently attainable technology.

There are a few places on Earth where lightning strikes are frequent. See **Table** **12** for a list of each continent’s ten most frequent locations for lightning.^[^ ^72^ ^]^ These data were recorded via a satellite instrument on the NASA Tropical Rainfall Measuring Mission. Assuming lightning power of ≈70 MW and duration of 0.5 s, available energies range from 2.3 to 0.5 kWh (mean value 1.0 kWh) for these top sites, neglecting losses.

**Table 12:** Locations of most frequent lightning arranged by continent (data from Albrecht et al.^[^ ^72^ ^]^)

Continent	Global rank	Flash rate [km^−2^ yr^−1^]	Lat [°]	Lon [°]	Place name	Country	Distance to place [km]
South America	1	232.52	9.75	−71.65	Lake Maracaibo (Lagunillas)	Venezuela	60.1
	4	172.29	7.55	−75.35	Cáceres	Colombia	3.4
	7	138.61	8.85	−73.05	El Tarra	Colombia	30.9
	11	124.26	5.75	−74.95	Norcasia	Colombia	20.4
	18	114.19	8.45	−74.55	Majagual	Colombia	12.6
	25	105.73	8.15	−76.85	Turbo	Colombia	14.8
	46	95.38	11.15	−72.95	Barrancas	Colombia	27.8
	74	87.96	−17.25	−65.05	Chimoré	Bolivia	34.9
	78	87.61	10.35	−70.95	El Corozo	Venezuela	27.5
	136	77.02	10.45	−75.35	Santa Rosa	Colombia	2.2
Africa	2	205.31	−1.85	27.75	Kabare	Democratic Republic of Congo	136.2
	3	176.71	−3.05	27.65	Kampene	Democratic Republic of Congo	124.9
	5	143.21	−0.95	27.95	Sake	Democratic Republic of Congo	140.0
	8	129.58	5.25	9.35	Nguti	Cameroon	11.7
	9	129.50	0.25	28.45	Butembo	Democratic Republic of Congo	94.3
	10	127.52	−1.55	20.95	Boende	Democratic Republic of Congo	141.2
	14	117.98	0.55	20.35	Boende	Democratic Republic of Congo	109.7
	15	117.19	−2.45	26.95	Kindu	Democratic Republic of Congo	126.7
	16	116.78	6.95	10.45	Baissa	Nigeria	36.6
	19	112.17	0.35	26.65	Kisangani	Democratic Republic of Congo	163.3
Asia	6	143.11	34.45	72.35	Daggar	Pakistan	14.0
	12	121.41	33.35	74.55	Rajauri	India	22.6
	13	118.81	33.75	70.75	Doaba	Pakistan	36.2
	22	108.03	14.55	43.45	Al Ḩadiyah	Yemen	13.2
	28	104.59	33.85	73.25	Murree	Pakistan	14.5
	31	101.79	25.25	91.95	Cherrapunji	India	26.1
	42	97.02	4.75	103.05	Paka	Malaysia	44.7
	45	95.92	1.95	103.85	Kota Tinggi	Malaysia	24.2
	50	94.64	3.75	98.05	Tenggulun	Indonesia	27.3
	52	93.96	3.15	101.65	Kuala Lumpur	Malaysia	4.2
North America	17	116.76	14.35	−91.15	Patulul	Guatemala	7.6
	29	103.23	14.85	−92.05	Catarina	Guatemala	2.8
	33	100.63	22.35	−83.95	San Luis	Cuba	20.1
	34	100.24	18.55	−74.35	Chambellan	Haiti	4.0
	37	99.39	13.15	−87.25	San Jerónimo	Honduras	12.7
	39	98.22	22.35	−80.65	Rodas	Cuba	9.8
	40	98.06	21.75	−78.85	Venezuela	Cuba	5.8
	47	95.32	22.85	−82.15	Mañalich	Cuba	4.3
	82	86.96	22.25	−105.25	Rosamorada	Mexico	14.9
	90	85.78	18.15	−77.65	Balaclava	Jamaica	2.6
Oceania	61	92.15	−15.35	125.35	Derby	Australia	284.4
	83	86.75	−14.45	126.55	Kununurra	Australia	278.0
	228	65.11	−16.65	124.75	Derby	Australia	139.6
	308	59.69	−15.65	128.45	Kununurra	Australia	34.6
	316	59.19	− 4.75	142.95	Ambunti	Papua New Guinea	61.0
	327	58.57	−15.25	129.45	Kununurra	Australia	95.8
	355	57.13	−13.15	131.05	McMinns Lagoon	Australia	66.6
	381	55.57	−13.95	129.95	Darwin	Australia	191.6
	471	51.35	−19.15	137.85	Mount Isa	Australia	245.6
	477	51.22	−17.45	126.05	Halls Creek	Australia	191.6

Direct use of lightning is limited by this low energy output compared to typical local demands. Power plant construction would be for the sake of research and to highlight scientific or technical knowledge rather than for practical considerations. On the other hand, lightning discharge is a high power phenomenon with currents and voltages in a range that may be suitable for specialized technical uses.

### 3.2 Active Use of Lightning

As described above, lightning phenomena are high‐power but low‐energy due to their short duration. In addition, capacitor and battery storage of direct lightning capture run into time difficulties related to rapid charging demands. Supercapacitors (0.3–30 s) are slow to charge compared with capacitors (10^−6^–10^−3^ s) and even these are close to being insufficient to meet the demands of rapid lightning stroke rates.

Direct capture of lightning using transformers to step up or down the voltage or current makes the process more versatile. Transducers with exploding wires wrapped as the primary coil are possible.^[^ ^39a^ ^]^ See **Figure** **5**. The development of extremely high voltage with this process might be useful for specialty materials processing, or perhaps for the input to high‐power lasers or for fusion technology, so long as the processes involved are insensitive to the natural variations in power input that are inherent.

### 3.3 Materials Processing

Materials processing with lightning requires selecting a technology where rapid application of power is acceptable. In rapid application, processing may be incomplete. **Table** **13** highlights nine processes which are possible. These involve melting materials, reducing volumes, chemical transformation, or energy conversion.

**Table 13:** Industrial and experimental uses of plasma arc technology (rapid uses in bold; left column from Zaghloul et al.^[^ ^3b^ ^]^)

Can use plasma arc technology	Rapid
**Titanium scrap melting**	**X**
Coal gasification	
Ferro‐alloy production	
Molten steel ladle heater	
**Aluminum recovery from dross**	**X**
**Volume reduction of equipment**	**X**
Tundish heating for steel casting	
**Iron ore reduction**	**X**
**Biomass energy conversion**	**X**
Shale oil recovery	
**Zinc recovery**	**X**
**Chemical synthesis**	**X**
MgO refractory production	
Powder metal production	
Silicon metal production	
Incinerator ash vitrification	
Electric arc furnace dust vitrification	
**Glass melting**	**X**
Waste pyrolysis	
• **municipal**	**X**
• medical	
• asbestos	
• tires	
• hazardous/toxic	
• low‐level radioactive	

Melting metal or glass may be amenable to processing where the demands of energy are otherwise prohibitive. Yet this process may be of low efficiency. Reducing material volumes may also be of low efficiency owing to low frequency of occurrence of lightning events. Notwithstanding, lightning strikes may be important for producing specialty products. The large flux of electrons present in a strike promotes reduced‐valence forms. The rapid processing time will result in a glassy material, without sufficient time for crystalline structures to form. A pilot project to build a processing plant would be important for gathering data to assess these possibilities.

Lightning strikes also reduce material strength. Where landscapes need to be transformed on a massive scale, e.g., excavating a mountain pass for roadway construction, directed strikes may be used as a complement to blasting with explosives. Controlled explosives are already relatively efficient and transportation of materials for blasting is a robust technology, though lightning weakening of materials may be of some use. Obviously, further study is needed. Weakening of materials such as granite or limestone before blasting would make a useful topic of study. Sections of the project without directed strikes could serve as experimental control.

Energy conversion via rapid combustion of materials is also a possibility. Leavitt’s model for explosions of water (Figure 4) may be adapted.^[^ ^30^ ^]^ A spark gap is located inside a chamber filled with the combustible material and an impulse turbine converts the resulting explosion to electricity, as a conductor brings the strike inside to spark over. The kinetic energy is a product of energy in the lightning strike and of stored chemical‐bond energy in the combustion material. In his experiments, Leavitt noted loss of material (from steel electrodes) on ignition and concluded that this may account for additional kinetic energy he measured.

The emissions spectra of this energy conversion process depend on the material. High power processes tend to break chemical bonds more effectively, and produce reduced (rather than oxidized) forms. Current plasma arc technologies for waste processing are expensive, and successful conversion via lightning energy is a reasonable basis for a pilot facility.

**Table** **14** summarizes the results from this section. Implementation of most processes listed depend on economic features, i.e., whether processing via lightning results in significant cost savings. Specialty‐materials production is important mainly for researching novel materials. In summary, triggered lightning might be used for materials processing in the place of a plasma arc, though further study is mandated since many of these processes currently use sustained power over tens of minutes or longer.

**Table 14:** Feasible material uses of lightning

Process	Examples	Benefits	Challenges
Metal preprocessing Glass preprocessing	Titanium scrap melting Aluminum recovery from dross Iron ore reduction Zinc recovery Glass melting	Lower cost	Material loss Incomplete processing Variable timing
Specialty material production	Glass metal alloy production	Novel materials and research data	Unknown
Landscape change	Granite weakening for road‐building blasting	Lower cost Increased speed	Unquantified effect Variable timing Personal danger
Waste processing Biomass energy production	Volume reduction of waste Municipal waste pyrolysis Conversion of switchgrass to syngas	Lower cost	Incomplete processing Variable timing Variable products

### 3.4 Managing the Strike

Those unfamiliar with triggered lightning will be surprised by how robust the technology is. Rocketry with a conducting tether is a consistent method of triggering lightning.^[^ ^2a,c,d^ ^]^ The technology is now used solely for study of lightning physics but may be adapted for industrial use. Triggering is offset by the expense of the rocket and tether.

For opening a plasma channel where desired, familiarity with exploding wires may help, especially to adapt lightning strikes for applications that need precise placement of a plasma channel. Another technology, laser triggering of lightning, has not been consistently successful to date, and requires high power outputs.^[^ ^73^ ^]^

### 3.5 Passive Use of Atmospheric Electricity

As stated earlier, natural variations are present in lightning phenomena. In addition to materials processing, these variations suggest passive harvesting of atmospheric electricity using modifications to current infrastructure. Energy capture from lightning protection systems is counter indicated in any condition where it will degrade the ability of the protection system to function properly.

As a pilot, during periods when there are no storms, lightning protection systems on structures can be used to harness fair weather electricity. An automated weather detection system might be used to determine when use is indicated, so long as its function is robust. During periods of thunderstorm activity, so long as impedance is low (≤5Ω), induction may be used to harness a fraction of the energy in a lightning discharge that is being transmitted via a lightning rod. Care should be taken that the system conforms with standards for lightning protection.

In agricultural areas, modest gains in ambient electricity might be integrated into a system that allow for agricultural usage. Pulsed electrical energy increases biomass production, likely acting to increase bioavailability of soil nutrients. Urban agriculture is suggested as a proper setting for further study, owing to the abundance of buildings where atmospheric electricity might be harvested. Even if agricultural applications are not desired, the modest increase in efficiency may be attempted so long as the infrastructure cost is minimal.

### 3.6 Other Modest Harvesting

A heat transducer does not produce much energy per strike, about 7.5 × 10^4^ J, yet could take the form of lightning rod pipes that produce energy via joule heating of water inside.^[^ ^21f^ ^]^ The water vapor is then run through an impulse steam turbine. Radiofrequency (RF) energy from thunderstorms are another possible source of energy for harvesting.^[^ ^36^ ^]^ Likewise, piezoelectric materials may harvest a small amount of energy from thunder. None of these will likely offset the materials cost involved in setting up a system, but research data may be useful if there is an interest in improving efficiencies in the built environment or for specialized (or miniature) applications.

### 3.7 Models for New Energy Production

In addition to the discussion above highlighting some modest practical applications, this study has produced two ideas for experimental power plant design that warrant further study. Both of these use natural processes as a model for generating electricity.

#### 3.7.1 Particle Collisions for Charge Separation

Thunderstorm clouds are active in creating high power charge separation via the behavior of large and small ice particles with each other and with supercooled water. These interactions include both collisions and induction. Thunderstorms persist on a scale of tens of hours to days and are active in translating a portion of the kinetic energy of wind into electrical energy.

The kinetic energies involved in generating the structure of a thunderstorm are immense, but the electricity itself of a thunderstorm is developed from the interactions of ice and water. The kinetic energy is harvested from the interactions of wind and particles as a natural charge separator. High electrical energy is present (i.e., many tens of megaelectronvolts) during the dart leader phase, where the production of runaway electrons is an important feature.^[^ ^2b^ ^]^ Likewise, high flux rates of low‐energy neutron emission occurs (3–5 × 10^−2^ neutrons cm^−2^ s^−1^) in thunderstorms as a result of low energy gamma rays produced during charge build‐up interacting with electrons via the photonuclear effect.^[^ ^44a^ ^]^ See **Table** **15** for typical kinetic and electric energies of thunderstorms.

**Table 15:** Kinetic and electric energies typically present in thunderstorms

Quantity	Energy (standard)	Energy (engineering)	Author(s)
Storm kinetic energy	2 × 10^6^ J m^−2^ 2 × 10^14^ J for 25 km diameter storm	50 GWh	Fuelberg and Scoggins (1978)^[^ ^74a^ ^]^
Storm electric energy • dissipation from lightning	10^10^–10^12^ J 10^9^–10^10^ J per flash	0.01–1 GWh	Mareev and Anisimov (2009)^[^ ^74b^ ^]^

Note that the Earth’s atmospheric electric circuit has varied in power over time, since the size and frequency of thunderstorms and precipitation as well as the conductivity of the atmosphere can change. Thus, thunderstorms as we know them now are only one instantiation of the process of charge separation via wind and materials collision interactions.

Volcanic lightning also relies on wind and material interactions. Fractoemission of electrons during particle collision produce both polarities of lightning as a feature of eruptive events.^[^ ^75^ ^]^ Lightning discharges during volcanic events are also a source of both RF and ground‐based electrical signals that occur during eruption.

**Figure 6:**
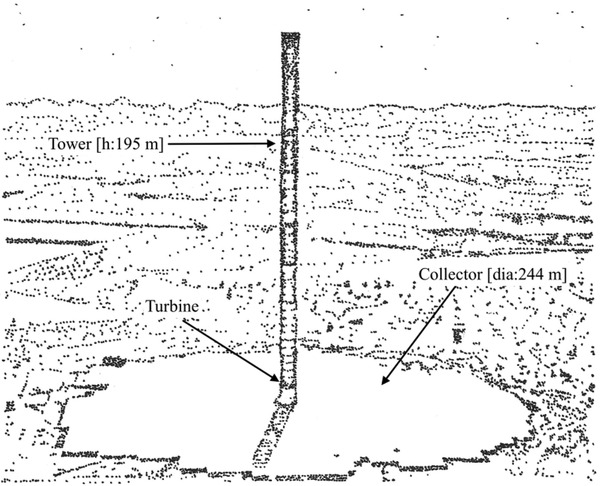
Solar updraft tower (50 kW). Manzanares, Spain. Operational 1982–1989. Image redrawn based on the work of Grose.^[^ ^76^ ^]^

In short, thunderstorms and volcanic eruptions are both members of a class of natural electricity generators that rely on wind and material interactions to create charge separation. A design for a charge separator based on the presence of wind and particles might be called a particle collision generator. At a minimum it ought to be located in a region that has ambient wind energy, or be based on a design to generate wind, e.g., a solar updraft tower that utilizes incoming solar radiation, and has a chamber wherein particles can interact, as well as electrodes to collect charge. See **Figure** **6** for an example of a solar updraft tower from Manzanares, Spain. The 46 ha collector is raised off of the ground and allows solar energy to heat air which exits via the tower with a turbine inside. A particle‐collision generator would forego turbines in favor of particles which collide and provide charge separation. If ice is meant to be one of the interacting materials, a generator may be built in regions with cold ambient temperatures. A system that combines particle‐collision charge separation with a solar updraft tower might improve the energy efficiency of the latter.

A simple particle‐collision electricity generator has the benefit of not requiring the mining of rare earth elements for turbine magnets. Like an ion‐wind generator, which works by pushing ions through a stable electric field, there are no large moving parts to monitor for strain.

This is a concept. No prototype has been built, and it is likely that power generation would be modest unless large windspeeds are attained. Other unknowns include the composition of the particles, the geometry of the chamber, and the functional location of electrodes, as well as a mechanism for particle containment if these may present an environmental or structural hazard. See **Table** **16** for a summary of critical unknowns related to the design of a particle‐collision generator.

**Table 16:** Critical unknowns for a particle‐collision electricity generator

Process	Unknown
Energy output	Quantification
Electrodes	Where to place Circuit with ground vs separate
Particle materials	Composition • ice • volcanic ash • other, e.g., lignin, quartz • mixed combinations Size Supply
Wind velocity	Optimization
Temperature	Optimization
Chamber design	Open vs closed Height Chimney/chamber diameter
Hybrid system	Damage to turbine Damage to environment

#### 3.7.2 Dusty Plasma Fusion Reactor

Ball lightning is a dusty or grain plasma. Fusion of lighter elements likely occurs inside ball lightning. More work could be done to explore artificial ball lightning formation and properties, especially what lends it stability, what forms its shell and how different chemistries of included material influence its properties. **Table** **17** summarizes critical unknowns in a plasma fusion reactor based on ball lightning.

**Table 17:** Critical unknowns for a ball lightning electricity generator

Process	Unknown	Utility
Plasma parameters	Target temperature Target energy Geometry of initiation Initiation duration	Nuclear fusion initiation Energy production Stability
Particle parameters	Composition • carbon • soil minerals • metals • gas components • (N_2_, H_2_O, O_2_, Ar, CO_2_, etc.) Size	Shell development Nuclear fusion initiation Nuclear fusion duration Energy production Stability Longevity Shape Color
Equipment parameters	Design Material composition	Generate electricity Prevent damage

### 3.8 Energy for Space Development

Space exploration and space development are nascent areas of public and scientific interest. Three features of this review are particularly important for energy generation in space. One involves the nature of charge separation in thunderstorms, and the other two are related to the electrical potential in the ionosphere.

First, the activity of charge separation in clouds described here is unique to Earth’s planetary environment. Saunders describes alternative mechanisms that are not the primary driver of charge separation in storms on Earth but may be useful in space where different parameters are active.^[^ ^5^ ^]^ Charge separation processes listed include droplet breakup; ion charging; convection; inductive charging; water splash freezing; accretion potential; lattice dislocations; temperature gradient; melting effects; ice splinter; and fragmentation. These each have different durations and magnitudes.

Second, the tether system described in Ogram for charging in the atmosphere is based on earlier work by NASA developing a tether system for ionospheric use.^[^ ^14^ ^]^ For example, an experimental space tether system for electricity generation was deployed in 1996, and used 0.5 km of conducting tether to generate 3.5 kV. The tether failed after five hours due to a spark discharge and material weakening.^[^ ^77^ ^]^ Magnetic and ionospheric interactions transmit energy.

Third, a nascent technology, space‐based solar energy, will transmit a microwave beam of energy from collecting satellites through the ionosphere to the ground. The transmission process is hindered by lack of an assigned frequency, as communications would be disrupted, but also may be problematic for creating traveling ionospheric disturbances (TID). These are associated with earthquake phenomena.^[^ ^78^ ^]^ TID can couple with conductive material in the Earth’s crust, e.g., ion‐laden water flow in faults, and may trigger earthquakes via magnetic field weakening of earth materials. This is the author’s proposed mechanism. There is no consensus in the research community for a reasonable mechanism. Notwithstanding, energy technologies involving the ionosphere will need to be associated with careful monitoring of seismic effects.

### 3.9 Lightning for Art

The earlier section on archaeology highlights human use of lightning strikes for cultural and religious reasons. A modern structure that is meant to be struck repeatedly might form the basis of a tourist destination, and could be a defining feature of a place that is forward‐facing. A public display is possible, but limited both by weather constraints and to locales with ample occurrence of lightning, i.e., with mountaintops nearby.

Likewise, using lightning to make art such as tapestries or sculptures with images from the strike burned into them may enjoy some public acclaim. The impact of these can serve to highlight technological advancement in conjunction with natural processes, and can help people to celebrate their values.

## 4 Conclusion

Total power of the Earth’s atmospheric electric circuit is about 250–500 MW. This is not enough power to justify large‐scale technological use of atmospheric electricity. Likewise, lightning phenomena are high‐power but low‐energy due to their short duration. Direct capture of lightning via tethered rocketry plus the use of an exploding wire transformer to step up or down the voltage or current makes the process more versatile if lightning discharge is to be used for specialized purposes or for material processing. This is more reasonable than capacitor, supercapacitor or battery storage owing to their slow charge times compared to lightning stroke duration. Specialized uses are also limited by lightning’s very short duration, and include processes that are amenable to natural variation, and that can benefit from cost reduction, for example, preprocessing of metals or glass, creation of novel research materials, waste volume reduction, biomass or waste energy transduction, as well as electrical weakening of mountain rock in preparation to blasting. Passive use of atmospheric electricity includes energy capture from lightning protection systems during fair weather conditions, or induction during storms so long as impedance is ≤5 Ω. Harvested energy is modest and may be used for promoting biomass growth, for example, in urban agriculture. These all serve to improve the efficiency of societal energy use but are likely not large enough to be transformative.

Additional uses may be more important during space development or in other planetary environments. Likewise, thunderstorm processes may inspire a novel particle‐collision electricity generator; and ball lightning may provide additional impetus for dusty plasma research and energy applications. Finally, lightning is an inspiring link between art and science, and can help to highlight technological progress. Lightning discharge is closely related to plasma physics research, and the field is still open to novel lines of experimentation and observation.

## 5 Experimental Section

This project was conducted during two distinct periods. During the course of two weeks in the middle of 2014, the academic database Google Scholar was searched using keywords related to ideas for practical applications of lightning, including as an energy source. Any time a new relevant paper was discovered by the author, keywords from the paper were noted and used for further searches. The search was continued until all the relevant fields that were identified had been searched through up to and including the final page accessible of the search, generally around 85 pages of ten results each. Search terms included: lightning manufacture; lightning fusion; lightning material; lightning fabrication; using lightning; Rogowski coil lightning; supercapacitor lightning; electromechanical energy lightning; harvesting lightning; as well as a few other basic searches initially made whose keywords were not recorded.

## Conflict of Interest

The author declares no conflict of interest.
